# Associations between Free Sugar and Sugary Beverage Intake in Early Childhood and Adult NAFLD in a Population-Based UK Cohort

**DOI:** 10.3390/children8040290

**Published:** 2021-04-08

**Authors:** Ahlia Sekkarie, Jean A. Welsh, Kate Northstone, Aryeh D. Stein, Usha Ramakrishnan, Miriam B. Vos

**Affiliations:** 1 Nutrition and Health Sciences Program, Laney Graduate School, Emory University, Atlanta, GA 30322, USA; jwelsh1@emory.edu (J.A.W.); aryeh.stein@emory.edu (A.D.S.); mvos@emory.edu (M.B.V.); 2 Department of Pediatrics, Emory School of Medicine, Atlanta, GA 30322, USA; 3 Population Health Science, Bristol Medical School, Bristol BS8 2BN, UK; kate.northstone@bristol.ac.uk; 4 Hubert Department of Global Health, Rollins School of Public Health, Emory University, Atlanta, GA 30322, USA; uramakr@emory.edu

**Keywords:** NAFLD, obesity, free sugars, sugar sweetened beverages, children, longitudinal, ALSPAC

## Abstract

(1) Background: High sugar intake is prevalent among children and is associated with non-alcoholic fatty liver disease (NAFLD). The purpose of this study is to determine if a high intake of free sugars and sugary beverages (SB) in childhood is associated with NAFLD in adulthood; (2) Methods: At 24 years, 3095 participants were assessed for severe hepatic steatosis (controlled attenuation parameter >280 dB/m) and had dietary data collected via a food frequency questionnaire at age three years. Multiple logistic regression models adjusted for total energy intake, potential confounders, and a mediator (offspring body mass index (BMI) at 24 years); (3) Results: Per quintile increase of free sugar intake association with severe hepatic steatosis at 24 years after adjusting for total energy was odds ratio (OR):1.07 (95% CL: 0.99–1.17). Comparing the lowest vs. the highest free sugar consumers, the association was OR:1.28 (95% CL: 0.88–1.85) and 1.14 (0.72, 1.82) after full adjustment. The OR for high SB consumption (>2/day) compared to <1/day was 1.23 (95% CL: 0.82–1.84) and OR: 0.98 (95% CL: 0.60–1.60) after full adjustment; (4) Conclusions: High free sugar and SB intake at three years were positively but weakly associated with severe hepatic steatosis at 24 years. These associations were completely attenuated after adjusting for confounders and 24-year BMI.

## 1. Introduction

The prevalence of non-alcoholic fatty liver disease (NAFLD), signified by elevated liver fat, has increased in parallel with the rise of obesity in children over recent decades, but has not received similar attention [1]. Pediatric NAFLD is particularly concerning because it can lead to inflammation, cirrhosis, and end-stage liver disease [2]. NAFLD is also linked to an increased risk of cardiovascular disease and diabetes [3,4]. Older age, male sex, obesity, and high sugar diets are some established risk factors for NAFLD [5].

Added sugars are sugars that are added to foods during processing [6]. Free sugars, which also include sugars that are naturally present in honey, syrups, fruit juices and fruit juice concentrate, are the primary sugars of public health concern because of their high prevalence in human diets [6]. Sugar-sweetened beverages (SSBs), which include all beverages with added sugars, and sugary beverages (SBs), which also include fruit juices, are the largest source of free sugars in children [7]. The World Health Organization (WHO) dietary guidelines for children and adults recommend limiting free sugar intake to less than 10% of daily energy to reduce the risk of overweight and obesity, and a further reduction to below 5% for additional health benefits [6]. The WHO also considers SSBs a “probable contributor” to the obesity epidemic [8]. Liquids are less satiating causing more postprandial hunger, therefore leading to increased energy intake [9]. SBs are also high in fructose, which directly contributes to the development of hepatic fat. The Scientific Advisory Committee on Nutrition (SACN) in the United Kingdom (UK) recommends that less than 5% of total energy intake comes from free sugars [10]. In the UK, according to the national diet and nutrition survey (NDNS), years 2014–2016, children 1.5 to 3 years old consume an average of 32.6 g of sugar a day, comprising 11.3% of their total energy intake [11]. Only 13% had intakes below or equal to 5% of total energy [11]. In the Avon Longitudinal Study of Parents and Children (ALSPAC), a UK-based birth cohort, the largest increase in intake of free sugars occurred during the preschool period when children increased their intake of free sugars from 12.3% at age 1.5 years to 16.4% of total energy at age 3.5 years [12]. In the NDNS, sugary beverages contributed 21% of free sugar consumption in children aged 1.5 to 3 years [11].

A high intake of dietary fructose is associated with NAFLD [13,14]. Compared to individuals of the same age without NAFLD, children and adults with NAFLD have a higher mean fructose intake, primarily due to higher consumption of soft drinks and fruit juices [15]. Reducing free sugar intake also can lead to a decrease in hepatic fat [16]. Due to the association between high sugar intake and NAFLD and the high prevalence of sugar intake in children, the strongest recommendation for treatment of NAFLD is to reduce sugar-sweetened beverage consumption [5]. Since dietary behavior, including sugar intake, can be modified and dietary patterns in early childhood can track into adulthood, identifying the most important modifiable factors in early life is an important area of research to improve outcomes for children [17]. Additionally, many children present with NAFLD beginning at puberty, so it is important to find preventive measures prior to the development of the disease [5].

The primary aim of this study is to describe intake of free sugar and sugary beverage intake in three-year-old children in the ALSPAC cohort and determine whether this intake is associated with hepatic steatosis in early adulthood (24 years of age).

## 2. Materials and Methods

### 2.1. Study Population

We used data from the Avon Longitudinal Study of Parents and Children, a population-based birth cohort study that has previously been described in detail [18,19,20]. In summary, ALSPAC enrolled 14,541 pregnant women in the Greater Bristol, UK area with expected delivery dates between 1 April 1991 and 31 December 1992. When the oldest children were seven years of age, attempts were made to increase the initial sample by recruiting from children that would have been eligible to enroll in the original study but did not join at the time, resulting in a new total of 15,454 pregnancies and 14,901 children. Clinical, dietary, and demographic information was collected throughout infancy and childhood. The first food frequency questionnaire (FFQ) was conducted at three years of age. At 24 years of age, 10,018 participants were invited to the Focus@24 clinic visit, during which clinic staff collected biological samples and took anthropometric measures. The Research Electronic Data Capture (REDCAP) tool hosted at the University of Bristol was used to collect and manage data from the 24-year clinic [21,22]. All of the study data are available on the study website, which also includes a fully searchable data dictionary and variable search tool [23].

The ALSPAC Ethics and Law Committee provided ethical approval for the study, and the data from the 24-year clinic was approved by the National Research Ethics Service Committee South West—Frenchay: 14/SW/1173 ALSPAC Focus at 24 + (24 February 2015, confirmed 20 March 2015).

### 2.2. Assessment of Free Sugar and Sugary Beverage Intake at Three Years of Age

Non-milk extrinsic sugars (NMES, or free sugars) as a percentage of total energy was calculated from responses to a non-quantitative FFQ, which aimed to cover all the primary foods consumed in Britain at the time and was completed by the mother. The FFQ included questions on weekly consumption frequency (never or rarely, once in two weeks, one to three times per week, four to seven times per week, and more than once a day) of 52 food groups and items [24]. Since portion sizes were not collected as part of the FFQ, standard portion sizes for children were assumed for nutrient estimates [25]. Nutrient intakes were calculated by multiplying the frequency of each food with the nutrient content of a portion of food [26]. NMES were calculated by deducting the sugars from milk, fruits, and vegetables from total sugars [12]. We then divided the NMES by total energy intake and categorized them into quintiles, with the lowest quintile as the reference.

The sugary beverage intake per day was quantified from mothers’ responses about their child’s weekly intake of sugary beverages. The number of sugary beverages consumed per day was included as a continuous measure and categorized [<1/day (reference group), 1 to 2/day, and >2/day] [27,28]. SBs included pure fruit juice, tinned juice, fruit drinks, Ribena™, squash, non-diet colas, and other “fizzy drink” questions. We did not include tea, coffee, and alcohol intake. Tea and coffee intake were negligible: Of the 30% that consumed tea at least once a day, only 10% added sugar. Only 7% reported at least one coffee a day, and of those, 7.3% reported adding sugar.

### 2.3. Assessment of Liver Outcomes

Participants’ liver steatosis and fibrosis were non-invasively assessed by transient elastography (FibroScan^®^ 502 Touch, Echosens, Paris, France) at 24 years of age. Transient elastography quantifies steatosis by providing a controlled attenuation parameter (CAP) measure. Exclusions from the scan included having an active medical implant such as a pacemaker, liver ascites, or being pregnant (*n* = 144). Prior to transient elastography, participants were asked to fast overnight or for at least six hours [29]. The Medium (M) or Extra Large (XL) probe was used to conduct the scan based on manufacturer and machine indications. A valid CAP score required ten readings for each participant. To be considered valid, CAP values also had to be within 100–400 dB/m. Invalid values were coded as missing.

Participants were categorized into two groups based on CAP score cut-off values derived from a meta-analysis by Karlas et al.: low to moderate (<280 dB/m, <66% steatosis) vs. severe steatosis (≥280 dB/m, ≥66% steatosis) [30]. For sensitivity analysis, we categorized CAP scores as low (<248 dB/m, <10% steatosis) vs. mild to severe hepatic steatosis.

### 2.4. Covariates

Offspring sex was extracted from medical records. The highest level of maternal education, which was self-reported during pregnancy, was used as a proxy for socioeconomic status [31]. Maternal education was categorized as follows: None/CSE (certificate of secondary education), Vocational (vocational courses after 16 years of age), O (ordinary level exams at 16 years), A (advanced level exams at 18 years), or University degree and above [32]. Mothers reported breastfeeding duration when their children were 15 months (never, <3 months, 3–6 months, or >6 months). At 24 years, weight and height were measured by trained study staff at the Focus@24 clinic. Body mass index (BMI) at 24 years was calculated from weight in kilograms divided by height in meters squared [33]. BMI at 24 years was then categorized as underweight (<18.5 kg/m^2^), normal weight (18.5 to <25 kg/m^2^), overweight (25 to <30 kg/m^2^), or obese (≥30 kg/m^2^) [34]. We adjusted for hazardous alcohol consumption using the Alcohol Use Disorder Identification Test for Consumption (AUDIT-C) score ≥4 in women and ≥5 in men [35,36].

### 2.5. Inclusion/Exclusion

We excluded individuals missing dietary information at three years and those missing a hepatic steatosis measure at 24 years.

### 2.6. Statistical Analysis

Statistical Analysis System (SAS) version 9.4 was used for statistical analyses (Cary, NC, USA). We calculated median and interquartile ranges (IQR) values for continuous variables and counts and percentages for categorical variables for the full sample and stratified by sugar intake quintile. We used F-tests to compare the differences in continuous variables and chi-squared tests to compare differences in categorical variables.

We used multiple logistic regression to model our associations between each exposure (free sugar percent quintiles and sugary beverage intake) and hepatic steatosis at 24 years. Free sugar percent quintiles were considered categorically in models to assess pairwise comparisons between those with the lowest intake (Q1) and highest intake (Q5). In separate models, the free sugar percent quintile was considered as a continuous term to assess the overall trend of increasing free sugar intake. The sugary beverage exposure was considered continuously and categorically as described above. In the base model, we adjusted for total energy intake. The second model adjusted for confounders including, the offspring sex, maternal education, maternal pre-pregnancy BMI, and duration of breastfeeding. The third model adjusted for the BMI category at 24 years as a potential mediator. In a fourth model, we adjusted for AUDIT-C score since alcohol intake is strongly associated with hepatic steatosis. Finally, in a sensitivity analysis, to further understand the role of total energy intake as a possible mediator in the association between a diet high in free sugars and hepatic steatosis, we compared models with and without total energy adjustment. All results were interpreted based on the size, direction, and confidence limits of the effect estimates and were not focused on statistical significance [37].

## 3. Results

**Figure 1:**
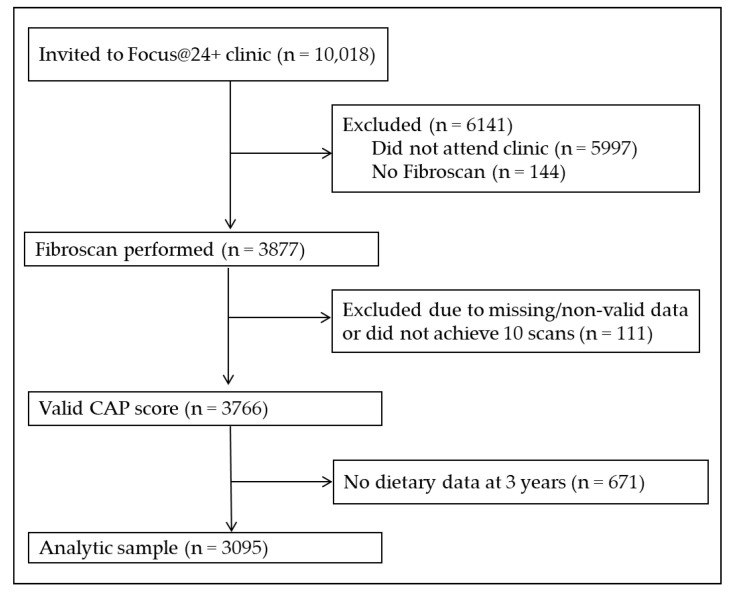
Participant flowchart for the analytic sample. Participants that did not attend the 24-year clinic or get a liver scan because they were ineligible due to an active implant, liver ascites, or pregnancy were excluded. Participants with missing or non-valid CAP scores or with missing dietary data from the three-year food frequency questionnaire were also excluded. Our final sample size was 3095.

The Focus@24+ clinic had 4021 of the 10,018 invited active ALSPAC participants attend, of which 3877 had FibroScan^®^ performed. Of these participants, 3766 had a valid CAP score. After exclusions for those missing dietary intake data at three years, our sample size was 3095 (Figure 1).

Table 1 presents sample characteristics by percent free sugar quintiles. Those in the lowest quintile (Q1) had free sugar intakes ranging from 0.14% to 11.5% of total energy (median = 28.5 g/day free sugars) and those in the highest quintile (Q5) had free sugar intakes ranging from 17.7% to 36.5% of total energy (median = 64.9 g/day free sugars). Less than 1% of participants met the level of free sugar intake recommended by the UK SACN (<5% of total energy). The median number of sugary beverages consumed per day was 1.4 (IQR: 0.9, 1.7). Approximately 17% consumed SBs less than once per day, and 21% consumed SBs more than twice a day. There was no association across quintiles of percent free sugar intake with child sex. Higher percent free sugar intake was associated with lower maternal education, shorter breastfeeding duration, higher total energy intake at three years, higher sugary beverage intake at three years, higher absolute sugar intake at three years, and hazardous alcohol use at 24 years.

**Figure 2:**
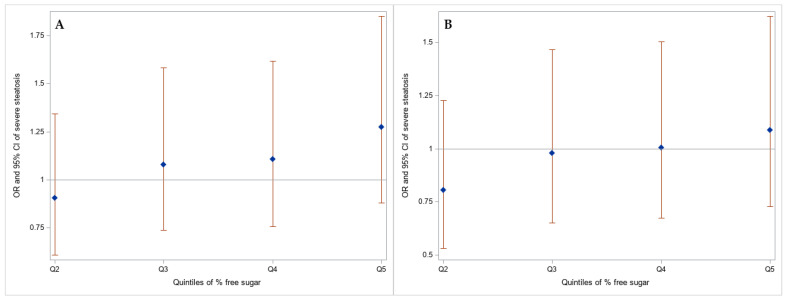
(**A**) Base (total energy intake) and (**B**) confounder (sex, maternal education, maternal pre-pregnancy body mass index, and breastfeeding duration) adjusted associations between percent free sugar of total energy intake quintiles (Ref = Q1) at three years and severe hepatic steatosis at 24 years in the ALSPAC cohort.

There was a positive but small and weak association [odds ratio (OR) 1.07, 95% CI: 0.99, 1.17] between increasing free sugar intake at three years and severe hepatic steatosis at 24 years (Table 2, Figure 2). Pairwise associations comparing those in the lowest free sugar intake quintile with higher levels of free sugar intake were generally positive, but these associations had wide confidence intervals (Table 2, Figure 2). For example, those consuming over 17.7% of their daily energy intake from free sugars at three years of age had 1.28 higher odds of severe hepatic steatosis at 24 years (95% CI: 0.88, 1.85). Adjusting for confounders and mediators did not meaningfully change the estimates.

The associations between continuous SB intake at three years and severe hepatic steatosis at 24 years were small and weak (Table 3). When SB intake was categorized, those consuming more SBs had higher odds of hepatic steatosis at 24 years compared to those consuming SBs less than once a day (>2 SB/day OR: 1.23, 95% CL: 0.82, 1.84) (Table 3). This association was attenuated after adjusting for confounders and BMI at 24 years (OR: 0.98, 95% CL: 0.60, 1.60), changing the primary outcome to mild-severe steatosis (as opposed to just severe steatosis) attenuated estimates (Table S1). When comparing models that did and did not adjust for total energy intake, estimates did not substantively change (Tables S2 and S3).

## 4. Discussion

We found that an increased intake of free sugars at three years of age had a small, positive association with severe hepatic steatosis at 24 years. There was also a weak positive association between high sugary beverage intake at three years of age and severe hepatic steatosis. This latter association was completely attenuated after controlling for the offspring BMI category at the time of outcome. A previous ALSPAC study looked at dietary intake at three years with hepatic steatosis at 17 years of age in a sub-sample of the overall study, although they did not specifically look at free sugars [38]. In that study, every 100 kcal increase in energy intake at three years of age (calculated from multi-level models that incorporated FFQ and food diary data) was associated with greater hepatic steatosis in adolescents (OR: 1.79, CL: 1.14–2.79). The food dietary data was available on only 10% of the sample. This association was mediated by fat mass in adolescence. There were no strong associations with any macronutrient intakes, including total sugar intake (38).

In other ALSPAC studies that looked specifically at sugary beverage intake, the primary outcome was adiposity, not hepatic steatosis. A previous analysis of sugar-sweetened beverage consumption in five- and seven-year-old children in the ALSPAC cohort found no evidence of an association with adiposity at age nine years [39]. However, that study did find a positive association between consumption of low-energy beverages in five- and seven-year-old children with adiposity at age nine years. The authors suggested that those at risk of obesity may be modifying their diets in an unsuccessful attempt to prevent obesity [39]. A separate ALSPAC study focused on central adiposity found that higher consumption of SSBs from 10 to 13 years of age was associated with a larger waist circumference at 13 years independent of total adiposity [31]. The results of these two studies are not necessarily in contrast since it has been shown that fructose, which is found in high amounts in many sugary beverages, specifically increases central adiposity [40,41]. 

Other studies have looked at the association between sugary beverages and hepatic fat but have not extended the outcome beyond childhood. In several cross-sectional studies, children with existing NAFLD had higher fructose and sucrose intake [42,43]. In the Generation R cohort, more than two sugary beverages per day compared to less than one per day at one year of age was associated with higher odds of MRI measured hepatic steatosis at 10 years old, independent of BMI at the time of outcome (OR: 1.34, 95% CL: 0.97, 1.83) [27]. We found similar associations, but in our study, they were mediated by BMI at the time of outcome. If the development of NAFLD requires obesity (particularly as individuals age), then the attenuation of estimates by adjusting for BMI is what we would expect to find, and the true association is what we derive from models not adjusting for BMI or adiposity.

The Generation R Cohort study also found that, compared to children with normal weight, children with overweight and obesity had stronger associations between SB intake at one year and mid-childhood steatosis. Compared to children with normal weight, overweight and obese children with NAFLD absorb and metabolize fructose more effectively [13,14]. Children susceptible to and with NAFLD have up-regulated de novo lipogenesis compared to non-NAFLD children [44].

The association between free sugar and sugary beverage consumption and hepatic steatosis is possibly partially mediated by overall energy intake. Therefore, adjusting for energy intake will lead to underestimates of the association between sugar exposures and hepatic steatosis [45,46]. We saw no differences between models with and without energy adjustment, indicating that any associations we found were not primarily mediated by total energy intake.

The largest strength of our study is that it utilizes a large, population-based longitudinal cohort from early childhood to 24 years of age. We also used a validated and accurate measure of hepatic steatosis, the CAP score based on transient elastography [30].

We did not exclude participants with other liver conditions since it has been previously reported that no participants in this cohort had or were taking medications for viral hepatitis, and very few were taking medications for autoimmune hepatitis [47]. Additionally, adjusting for hazardous alcohol intake did not change the estimates; therefore, we are confident that NAFLD is the primary cause of hepatic steatosis in our population.

One of the possible reasons that we did not see strong associations was that most participants had an intake of free sugar above recommended levels. Less than 1% of our sample had intakes within the recommended levels, and those in our reference group had free sugar intakes up to 11.5% of total energy. Additionally, there are limitations to the FFQ that was used to measure dietary intake. Portion sizes were not ascertained, and so calculated intakes may be inaccurate. Furthermore, there were other factors that contribute to hepatic steatosis that we were not able to adjust for, including genetics and lifestyle variables that extend from childhood to adulthood.

The participants of this cohort were primarily of white ethnicity, limiting the generalizability of this study to other populations. For example, there may be stronger associations in populations with individuals at a higher risk of NAFLD, such as Hispanics who have a higher proportion of individuals with adipogenic genes such as Patatin-like phospholipase domain-containing protein 3 (PNPLA3) [48,49]. Females and participants with mothers that had higher education were more likely to remain in the cohort leading to differential loss to follow-up.

## 5. Conclusions

Free sugar intake in three-year-old children, as measured in our study, was positively but weakly associated with hepatic steatosis in young adulthood. The positive association between high sugary beverage intake at three years and hepatic steatosis in young adulthood was mediated by BMI at the time of outcome. Children should continue to limit their intake of free sugars and sugary beverages. Further longitudinal studies with validated measures of sugar intake and hepatic steatosis throughout childhood are important.

## Figures and Tables

**Table 1:** Sample characteristics by percent free sugar of total energy intake quintile presented as *n* (%) or median (IQR) in the ALSPAC cohort.

Variable	Overall	Q1	Q2	Q3	Q4	Q5	*p*-Value
	N = 3095	(0.14%–11.5%)	(11.5%–13.5%)	(13.5%–15.3%)	(15.3%–17.7%)	(17.7%–36.5%)	
Free sugars (g) at 3 years	43.5 (33.6, 54.7)	28.5 (23.2, 33.6)	38.4 (32.5, 44.5)	43.6 (37.3, 50.3)	50.9 (43.9, 59.0)	64.9 (52.9, 78.3)	<0.001
TEI (kJ) at 3 years	5072 (4346, 5858)	4849 (4074, 5515)	5095 (4377, 5842)	5049 (4342, 5800)	5187 (4486, 6011)	5287 (4430, 6297)	<0.001
Sugary beverages/day	1.6 (1.3, 2.0)	1.4 (0.8, 1.6)	1.5 (1.1, 1.7)	1.6 (1.4, 1.9)	1.7 (1.4, 2.2)	1.9 (1.5, 2.4)	<0.001
Male sex	1200 (38.8%)	234 (37.8%)	241 (38.9%)	243 (39.3%)	250 (40.4%)	232 (37.5%)	0.839
Maternal education							
CSE/None	257 (8.3%)	46 (7.4%)	40 (6.5%)	47 (7.6%)	49 (7.9%)	75 (12.1%)	<0.001
Vocational	206 (6.7%)	43 (6.9%)	34 (5.5%)	33 (5.3%)	39 (6.3%)	57 (9.2%)	
O-level	1042 (33.7%)	180 (29.1%)	197 (31.8%)	193 (31.2%)	226 (36.5%)	246 (39.7%)	
A-level	895 (28.9%)	202 (32.6%)	194 (31.3%)	186 (30.0%)	170 (27.5%)	143 (23.1%)	
Degree	657 (21.2%)	140 (22.6%)	144 (23.3%)	153 (24.7%)	131 (21.2%)	89 (14.4%)	
Maternal BMI							
Underweight	117 (3.8%)	27 (4.4%)	32 (5.2%)	20 (3.2%)	18 (2.9%)	20 (3.2%)	0.09
Normal	2239 (72.3%)	442 (71.4%)	419 (67.7%)	474 (76.6%)	458 (74.0%)	446 (72.1%)	
Overweight	392 (12.7%)	68 (11.0%)	98 (15.8%)	65 (10.5%)	83 (13.4%)	78 (12.6%)	
Obese	122 (3.9%)	29 (4.7%)	26 (4.2%)	20 (3.2%)	21 (3.4%)	26 (4.2%)	
Breastfeeding duration							
Never	468 (15.1%)	84 (13.6%)	74 (12.0%)	80 (12.9%)	89 (14.4%)	141 (22.8%)	<0.001
<3 m	613 (19.8%)	113 (18.3%)	118 (19.1%)	121 (19.5%)	130 (21.0%)	131 (21.2%)	
3–5 m	512 (16.5%)	107 (17.3%)	105 (17.0%)	110 (17.8%)	116 (18.7%)	74 (12.0%)	
>6 m	1378 (44.5%)	284 (45.9%)	303 (48.9%)	288 (46.5%)	262 (42.3%)	241 (38.9%)	
AUDIT-C at 24 yrs	5 (4, 7)	5 (3, 7)	5 (4, 7)	5 (4, 7)	6 (4, 7)	5 (3, 7)	0.024
BMI at 24 yrs							
Underweight	90 (2.9%)	16 (2.6%)	19 (3.1%)	19 (3.1%)	18 (3.0%)	18 (2.9%)	
Normal	1846 (60.2%)	373 (61.3%)	359 (58.5%)	386 (62.6%)	365 (59.5%)	363 (59.2%)	0.7780
Overweight	762 (24.9%)	147 (24.4%)	156 (25.4%)	147 (23.8%)	159 (25.9%)	153 (25.0%)	
Obese	369 (12.0%)	73 (12.0%)	80 (13.0%)	65 (10.5%)	72 (11.7%)	79 (12.9%)	
Severe hepatic steatosis ^1^	304 (9.8%)	56 (9.0%)	52 (8.4%)	61 (9.9%)	63 (10.2%)	72 (11.6%)	0.378

^1^ Steatosis is defined from controlled attenuation parameter scores: severe (>279 dB/m). The following variables had missing values: sugary beverage intake (*n* = 7, 0.2%), maternal education (*n* = 38, 1.2%), maternal BMI (*n* = 225, 7.3%), breastfeeding (*n* = 124, 4.0%), BMI at 24 years (*n* = 28, 0.9%), and AUDIT-C score (*n* = 63, 2.0%). F-tests were used to compare differences in continuous variables and chi-squared tests were used to compare differences for categorical variables. Abbreviations: ALSPAC = Avon Longitudinal Study of Parents and Children, Q 1–Q 5 = quintiles 1 to 5, IQR = interquartile range, CSE = certificate of secondary education, TEI = total energy intake, yrs = years, AUDIT-C = Alcohol use disorder identification test–concise, BMI = body mass index, m = months.

**Table 2:** Percent free sugar of total energy intake quintiles at three years and severe hepatic steatosis at 24 years in the ALSPAC cohort.

		Q1 (0.14–11.5)	Q2 (11.5–13.5)			Q3 (13.5–15.3)			Q4 (15.3–17.7)			Q5 (17.7–36.6)			Per Quintile			*p*-Trend
Model	*n*	REF	OR	95% CL		OR	95% CL		OR	95% CL		OR	95% CL		OR	95% CL		
1	3095	1.00	0.90	0.61	1.34	1.08	0.74	1.58	1.11	0.76	1.62	1.28	0.88	1.85	1.07	0.99	1.17	0.103
2	2742	1.00	0.81	0.53	1.23	0.98	0.65	1.47	1.01	0.67	1.50	1.09	0.73	1.62	1.04	0.95	1.14	0.394
3	2715	1.00	0.67	0.41	1.08	1.04	0.65	1.66	0.99	0.62	1.58	1.14	0.72	1.82	1.07	0.96	1.19	0.204
4	2685	1.00	0.77	0.50	1.18	0.99	0.66	1.49	0.99	0.66	1.49	1.09	0.73	1.63	1.05	0.95	1.15	0.355

Model 1: adjusts for total energy intake. Model 2: model 1 + sex, maternal education, maternal pre-pregnancy body mass index, and breastfeeding duration. Model 3: model 2 + body mass index category at 24 years. Model 4: model 2 + AUDIT-C (Alcohol Use Disorder Identification Test–Concise) score at 24 years. Abbreviations: ALSPAC = Avon Longitudinal Study of Parents and Children, Q1–Q5 = quintiles 1 to 5, REF = reference, OR = odds ratio, CL = confidence limits.

**Table 3:** Sugary beverage (SB) intake per day at 3 years and severe hepatic steatosis at 24 years in the ALSPAC cohort.

		Continuous SB/Day			<1/Day	1–2/Day			>2/Day		
Model	*n*	OR	95% CL		REF	OR	95% CL		OR	95% CL	
1	3088	1.04	0.87	1.24	1.00	1.25	0.89	1.77	1.23	0.82	1.84
2	2739	1.04	0.86	1.25	1.00	1.18	0.81	1.70	1.19	0.77	1.83
3	2715	0.92	0.73	1.15	1.00	1.03	0.68	1.57	0.98	0.60	1.60
4	2682	1.03	0.85	1.25	1.00	1.19	0.82	1.73	1.20	0.78	1.86

Model 1: adjusts for total energy intake. Model 2: model 1 + sex, maternal education, maternal pre-pregnancy body mass index, and breastfeeding duration. Model 3: model 2 + body mass index category at 24 years. Model 4: model 2 + AUDIT-C (Alcohol Use Disorder Identification Test–Concise) score at 24 years.

## Data Availability

The data that support the findings of this study are available from the Avon Longitudinal Study of Parents and Children but restrictions apply to the availability of these data, which were used under license for the current study, and so are not publicly available. Data are however available from the authors upon reasonable request and with permission of ALSPAC. Researchers can apply to ALSPAC for use of the data. The study website (http://www.bristol.ac.uk/alspac/researchers/our-data/) (accessed on 07 April 2021) contains details of all the data that are available through a fully searchable data dictionary and variable search tool.

## Supplementary Materials

The following are available online at https://www.mdpi.com/article/10.3390/children8040290/s1, Table S1: Sugary beverage intake per day at 3 years and mild to severe hepatic steatosis at 24 years in the ALSPAC cohort; Table S2: Adjusted ^1^ associations between free sugar percent quintiles at 3 years and severe hepatic steatosis at 24 years with and without total energy intake (TEI) in the ALSPAC cohort; Table S3: Adjusted ^1^ associations between sugary beverage intake at 3 years and severe hepatic steatosis at 24 years with and without total energy intake (TEI) in the ALSPAC cohort.
