# Supplementary material for: Associations between Free Sugar and Sugary Beverage Intake in Early Childhood and Adult NAFLD in a Population-Based UK Cohort
Source: Children (Basel). 2021 Apr 8;8(4):290. doi: 10.3390/children8040290 (PMC8068295; doi:10.3390/children8040290)
Supplement: Supplementary file 1 [file children-08-00290-s001.pdf]

Supplementary

Table S1. Sugary beverage intake per day at 3 years and mild to severe hepatic steatosis at 24 years in the ALSPAC cohort.

		**continuous SB/day**			**<1/day**	**1–2/day**			**>2/day**		
**Model**	***n***	**OR**	**95% CL**		**REF**	**OR**	**95% CL**		**OR**	**95% CL**	
1	3088	1.02	0.89	1.16	1.00	0.99	0.78	1.26	0.95	0.71	1.26
2	2739	1.05	0.91	1.21	1.00	0.96	0.74	1.24	0.96	0.70	1.31
3	2715	0.99	0.83	1.17	1.00	0.84	0.62	1.13	0.81	0.57	1.16
4	2682	1.05	0.91	1.22	1.00	0.98	0.75	1.27	0.97	0.71	1.33

Model 1: adjusts for total energy intake. Model 2: model 1 + sex, maternal education, maternal pre-pregnancy body mass index, and breastfeeding duration. Model 3: model 2 + body mass index category at 24 years. Model 4: model 2 + AUDIT-C (Alcohol Use Disorder Identification Test – Concise) score at 24 years. Abbreviations: ALSPAC = Avon Longitudinal Study of Parents and Children, SB = sugary beverages, OR = odds ratio, CL = confidence limits.

Table S2. Adjusted [1] associations between free sugar percent quintiles at 3 years and severe hepatic steatosis at 24 years with and without total energy intake in the ALSPAC cohort.

		**Q1 (0.14–11.5)**	**Q2 (11.5–13.5)**			**Q3 (13.5–15.3)**			**Q4 (15.3–17.7)**			**Q5 (17.7–36.6)**			**Per quintile**			**p-trend**
	n	**REF**	**OR**	**95% CL**		**OR**	**95% CL**		**OR**	**95% CL**		**OR**	**95% CL**		**OR**	**95% CL**		
No TEI adjustment	2742	1.00	0.82	0.54	1.24	0.99	0.66	1.48	1.02	0.69	1.52	1.11	0.75	1.65	1.05	0.95	1.14	0.35
TEI adjustment	2742	1.00	0.81	0.53	1.23	0.98	0.65	1.47	1.01	0.67	1.50	1.09	0.73	1.62	1.04	0.95	1.14	0.39

[1] Both models are also adjusted for sex, maternal education, maternal pre-pregnancy body mass index, and breastfeeding duration. Abbreviations: ALSPAC = Avon Longitudinal Study of Parents and Children, TEI = total energy intake, OR = odds ratio, CL = confidence limits.

Table S3. Adjusted [1] associations between sugary beverage intake at 3 years and severe hepatic steatosis at 24 years with and without total energy intake in the ALSPAC cohort.

		**continuous SB/day**			**<1/day**	**1–2/day**			**>2/day**		
	n	**OR**	**95% CL**		**REF**	**OR**	**95% CL**		**OR**	**95% CL**	
NO TEI adjust-ment	2739	1.05	0.87	1.26	1.00	1.18	0.82	1.71	1.21	0.79	1.85

TEI adjustment	2739	1.04	0.86	1.25	1.00	1.18	0.81	1.70	1.19	0.77	1.83

[1] Both models are also adjusted for sex, maternal education, maternal pre-pregnancy body mass index, and breastfeeding duration. Abbreviations: ALSPAC = Avon Longitudinal Study of Parents and Children, SB = sugary beverages, TEI = total energy intake, OR = odds ratio, CL = confidence limits.
